# Factors Associated with High-Quality Cardiopulmonary Resuscitation Performed by Bystander

**DOI:** 10.1155/2020/8356201

**Published:** 2020-02-27

**Authors:** Hye Ji Park, Won Jung Jeong, Hyung Jun Moon, Gi Woon Kim, Jin Seong Cho, Kyoung Mi Lee, Hyuk Joong Choi, Yong Jin Park, Choung Ah Lee

**Affiliations:** ^1^Department of Emergency Medicine, Hallym University, Dongtan Sacred Heart Hospital, Hwaseong-si, Gyeonggi-do, Republic of Korea; ^2^Department of Emergency Medicine, Catholic University of Korea, St. Vincent's Hospital, Suwon, Gyeonggi-do, Republic of Korea; ^3^Department of Emergency Medicine, College of Medicine, Soonchunhyang University, Cheonan-si, Chungcheongnam-do, Republic of Korea; ^4^Department of Emergency Medicine, College of Medicine, Soonchunhyang University, Bucheon-si, Gyeonggi-do, Republic of Korea; ^5^Department of Emergency Medicine, Gil Medical Center, Gachon University College of Medicine, Incheon, Republic of Korea; ^6^Department of Emergency Medicine, Myongji Hospital, Goyangsi, Gyeonggo-do, Republic of Korea; ^7^Department of Emergency Medicine, Hanyang University Guri Hospital, Guri-si, Gyeonggo-do, Republic of Korea; ^8^Department of Emergency Medicine, Chosun University Hospital, Gwangju, Republic of Korea

## Abstract

Bystander cardiopulmonary dresuscitation (CPR) improves the survival and neurological outcomes of sudden cardiac arrest patients. The rate of bystander CPR is increasing; however, its performance quality has not been evaluated in detail. In this study, emergency medical technicians (EMTs) in the field evaluated bystander CPR quality, and we aimed to investigate the association between bystander information and CPR quality. This retrospective cohort study was based on data included in the Smart Advanced Life Support (SALS) registry between January 2016 and December 2017. We included patients older than 18 years who experienced an out-of-hospital cardiac arrest (OHCA) due to medical causes. Bystander CPR quality was judged to be “high” when the hand positions were appropriate and when compression rates of at least 100/min and compression depths of at least 5 cm were achieved. Among 6,769 eligible patients, 3,799 (58.7%) received bystander CPR, and 6% of bystanders performed high-quality CPR. After adjustment, the occurrence of cardiac arrest at home (adjusted odds ratio (aOR), 95% confidence interval (CI); 0.42, 0.27–0.64), witnessed cardiac arrest (1.45, 1.03–2.06), and younger bystander age all showed associations with one another. High-quality CPR led to a 4.29-fold increase in the chance of neurological recovery. In particular, high-quality CPR in patients aged 60 years showed a significant association compared with other age groups (7.61, 1.41–41.04). The main factor affecting CPR quality in this study was the age of the bystander, and older bystanders found it more difficult to maintain CPR quality. To improve the quality of bystander CPR, training among older bystanders should be the focus.

## 1. Introduction

Out-of-hospital cardiac arrest (OHCA) is a serious public health problem worldwide due to a high incidence and low survival rates [1, 2]. The role of the bystander is very important for improving survival. Bystander cardiopulmonary resuscitation (CPR) improves the survival and neurological outcomes of victims with sudden cardiac arrest [3–5]. To promote bystander CPR, we have augmented dispatcher-assisted CPR (DACPR) and CPR education programs for laypersons. Consequently, the rate of bystander CPR has increased [6]. In addition, many studies have indicated the importance of CPR quality. Gallagher et al. reported no difference in survival between cardiac arrest (CA) patients without adequate chest compressions and no chest compressions [7]. According to Yu et al., the quality of chest compression is a more important determinant of successful resuscitation than defibrillation or rapidity of chest compression [8]. However, the performance quality of bystander CPR has not been studied adequately. It is difficult to objectively evaluate the quality of bystander CPR in the field. Furthermore, there was no uniform format for evaluating these activities.

In this study, emergency medical technicians (EMTs) in the field evaluated bystander CPR quality, and we aimed to investigate the association between bystander information and CPR quality.

## 2. Methods

### 2.1. Study Design and Setting

This retrospective cohort study was based on data included in the Smart Advanced Life Support (SALS) registry between January 2016 and December 2017. This is a prospective, population-based registry of OHCA cases that occurred in 18 urban and suburban areas, encompassing a total area of 7129.49°km^2^ and a total population of 11.6 million inhabitants.

### 2.2. Study Populations

SALS is a method of advanced field resuscitation performed by paramedics under direct, video communication-based medical direction on patients with OHCA of medical causes older than 18 years in Korea [9, 10]. The exclusion criteria included the following: obvious signs of death, age under 18 years, refusal of CPR, do-not-resuscitate state, noncardiac origin of CA, and incomplete patient data. Additionally, patients in whom CPR was ceased immediately due to futility; who called on 911 by themselves; whose CA was witnessed by first responders or emergency medical services (EMS) personnel; or who had incomplete bystander data records were excluded.

### 2.3. Measurements

The quality of chest compressions was evaluated by EMTs on arrival at the scene. EMTs requested bystanders to continue their CPR and made a subjective assessment based on their short observation following arrival at the scene. They classified bystander CPR into two categories “high-quality” and “low-quality.” Bystander CPR quality was judged to be “high” when the hand positions were appropriate and compression rates of at least 100/min and compression depths of at least 5 cm were ensured [11]. We compared CPR-related factors and outcomes according to bystander CPR quality. According to the International Utstein style for CA [12], patient-related factors included patients' age, sex, and comorbidities. The event-related factors included location at the time of event, witness status, bystander CPR, and causes of CA. The system-related factors included quality of CPR, dispatcher-assisted telephone CPR, and EMS response time. We investigated bystander information: sex, age group (decade-years intervals), relation with victim, and number of bystanders. The relation with the patient was classified as family and nonfamily. Multirescuers were defined as 2 or more bystanders involved in resuscitation. The correlation between high-quality bystander CPR and resuscitation-related factors was investigated.

The outcomes of patients were measured as prehospital return of spontaneous circulation (ROSC), total ROSC, survival at discharge, and neurologically favorable discharge. A neurologically favorable discharge was defined as a hospital discharge with a cerebral performance category score of 1 or 2 [1]. Furthermore, the effect of high-quality CPR on the neurologic prognosis of patients was evaluated according to the age of the bystander as a secondary outcome.

### 2.4. Statistical Analysis

Statistical analyses were performed using SPSS, version 24.0 (IBM Corp., Armonk, NY, USA). The patient- and resuscitation-related characteristics and outcomes were compared between the high-quality and low-quality CPR groups. Chi-square and Mann-Whitney *U* tests were used to compare categorical and continuous variables, respectively.

To identify the factors associated with high-quality CPR, we applied regression analyses for factors that were significant in the univariate analyses. No multicollinearity was detected, and all relevant interactions were considered. Additionally, the analysis of factors influencing survival at discharge and a neurologically favorable discharge according to the age of the bystander included targeted temperature management as a confounder. A *p* value of <0.05 was considered statistically significant.

### 2.5. Ethical Statement

This study was approved by the Institutional Review Board at Hallym University (Approval number: HDT 2018-10-004), and the need for informed consent was waived.

## 3. Results

**Figure 1:**
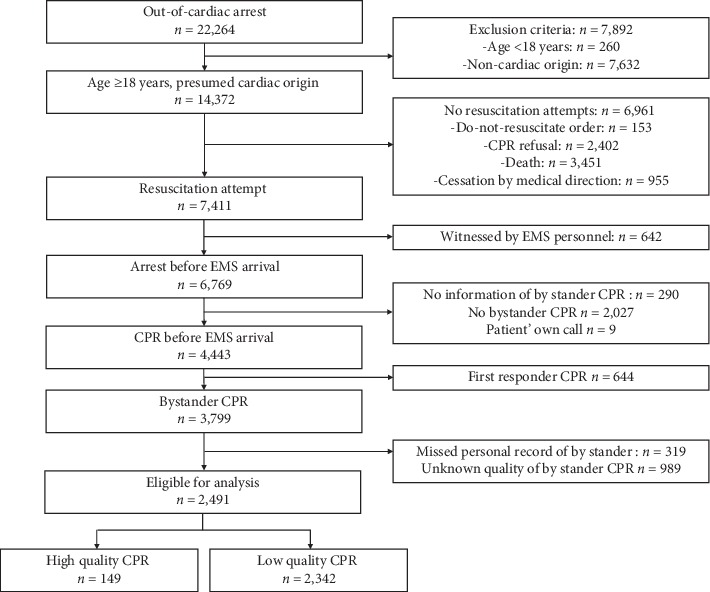
Study flow diagram.

Among 22,264 CA patients, 14,372 (64.6%) experienced a nontraumatic CA and were aged 18 years or above, and 7,411 underwent resuscitation. The number of patients who underwent resuscitation before the arrival of 1 EMS was 6,769, and among these, 3,799 (58.7%) received bystander CPR (10% received CPR by the first responder). After the exclusion of cases in which no personal information was available for the bystanders who provided CPR, 2,491 cases were analyzed (Figure 1).

### 3.1. General Patient Characteristics

There were 149 cases (6.0%) involving high-quality CPR. The number of high-quality CPR cases was higher for male patients (*n* = 115, 77.2%) (*p*=0.005) and younger patients (*p* < 0.001). The number of lower quality CPR cases was significantly higher in patients with hypertension, cerebrovascular disease, or heart disease. When high-quality CPR was maintained, the first rhythm after EMS arrival was shockable in 35.8% of the patients, which was higher than the percentage in low-quality CPR cases (*p* < 0.001). In addition, the high-quality CPR rate of CA at home was lower. The rate of high-quality CPR was significantly higher in witnessed CA cases (*p*=0.011).

### 3.2. General Bystander Characteristics

There was no difference in CPR quality according to sex, and the rate of high-quality CPR was higher in the younger age group (*p* < 0.001). When the CPR provider was a family member of the patient, the CPR quality was significantly lower (*p* < 0.001). There was no association between the CPR quality and the number of rescuers (*p*=1.000). There was no significant association between DACPR or the time interval of EMS response and the quality of bystander CPR. When high-quality bystander CPR was conducted, the rates of prehospital ROSC (41.6% vs. 22.5%), total ROSC (47.7% vs. 29.0%), survival at discharge (30.2% vs. 10.1%), and neurologically favorable discharge (25.5% vs. 5.7%) were all high (Table 1).

### 3.3. Factors Affecting High-Quality CPR

In the univariate analysis, high-quality CPR showed a significant association with the patient's age and sex, occurrence of CA at home, witnessed CA, and bystander factors including familiar relation. After adjustment, occurrence of CA at home (aOR, 95% confidence interval (CI); 0.42, 0.27–0.64), witnessed cardiac arrest (aOR, 95% CI; 1.45, 1.03–2.06), and younger age of the CPR provider all showed associations with high-quality CPR (Table 2).

### 3.4. Relationship of High-Quality CPR with Age Group and Patient Outcome

The rates of survival at discharge in all age groups and neurologically favorable discharge were 2.84- and 4.29-times higher, respectively, in the high-quality CPR group than in the low-quality CPR group. When high-quality CPR was conducted, the rate of neurologically favorable discharge was 7.61-times higher in patients aged 60 years or older than in the other age groups (Table 3).

## 4. Discussion

In the present study, we evaluated the quality of bystander CPR on a large scale using multiregional data and determined the association between bystander-related factors and the quality of CPR, as well as patient factors. As a result, young age of bystanders was associated with high-quality CPR. In addition, the quality of CPR performed by 60-year-old bystanders was the lowest; however, high-quality CPR performed by this group had a greater effect on the neurological recovery of patients.

Bystander CPR is considered a key factor affecting patient survival after OHCA [12]. However, the bystander CPR rate in 2006 was very low at 2.1% in Korea [13]. Since 2008, we have enacted the Good Samaritan Law and maintained the mandated first responder and school CPR programs. Since 2011, the DACPR system has been operated by the national EMS [6]. Therefore, the bystander CPR rate has increased dramatically, as seen in this study. However, little is known regarding the quality of CPR performed by bystanders. A study using CPR quality data stored by automated external defibrillators showed limitations including no bystander information and inclusion bias [14]. Assessment of simulated CPR could not reflect the situation of a real population [15, 16]. Takei et al. evaluated bystanders in a single area [11]. However, we determined the quality status of CPR and the bystander factors related to high-quality CPR in a large multiregional study.

### 4.1. Realities of Bystander CPR

The rate of bystander CPR (58.7%) in this study was very high compared to those in other studies in Asia (17.3%), France (19.4%), and Denmark (34.9%) [17–19]. All emergency calls in Korea are integrated into a public EMS call center that provides CPR instructions in all CA situations. As in a previous study, it was believed that dispatcher instruction significantly increased the actual provision of bystander CPR among adult OHCA patients [20].

When EMTs evaluated CPR performance of the bystanders on the scene, high-quality CPR accounted for only 6%, which was very low compared to that in a population-based study in Japan using the same measurement method (80.7%) [11]. This may be attributed to the lack of CPR education, and although DACPR increased B-CPR, it did not increase the quality but rather increased the rate of low-quality CPR.

### 4.2. Factors Affecting Bystander CPR Quality

As seen in previous studies, the sex and age of patients did not affect CPR quality [11]. Patients with hypertension, cerebrovascular disease, or heart disease were resuscitated with low-quality CPR. CPR education, which increases the CPR performance, has been encouraged in families of patients with cardiovascular disease but does not seem to induce an increase in quality.

In the case of CA occurring at home, the quality of bystander CPR was particularly low (aOR, 95% CI 0.42, 0.27–0.64). It has been reported that DACPR is especially beneficial for the initiation of bystander CPR in residential areas [19]. Eventually, we assumed that the rate of low-quality CPR increased at home.

Witnessed CA was significantly associated with high-quality CPR, differing from that reported in a previous study, which showed no association between these factors [11]. Witnessing the CA may have led to more active participation of the bystander in CPR.

There was no association between CPR quality and DACPR, emergency response time, and sex of the bystander, consistent with that in a previous study [11]. However, the number of rescuers also showed no correlation with the quality of CPR, inconsistent with that reported in a previous study, which reported that multiple rescuers resulted in a 2.27-times increase in the quality of CPR [11]. In the case of multirescuers, if one team member is proficient, then the number of people who are good at CPR may be inconsequential.

The main factor affecting CPR quality in this study was the age of the bystander. After adjusting for the patient's age and sex, location of arrest (home), witness status, and familial relation with the patient, the rate of high-quality CPR was 4.28 times higher in bystanders under 40 years of age than in those over 60 years of age. Takei et al. reported that high-quality CPR was not related to bystander age; however, a relationship between high-quality CPR and the younger age group was observed in our population. In a study on health-care providers, elderly providers (>65 years) showed decreased CPR quality compared to younger providers [15]. The elderly are generally physically weaker than younger people. In a 2008 study, the participants of a mandatory training program for CPR were students and first responders [6]. Retired elderly were likely to lack CPR knowledge because they had fewer educational opportunities. As a secondary outcome, high-quality CPR can lead to a 4.29-times increase in neurological recovery. In particular, high-quality CPR in patients aged 60 years and above had an aOR of 7.61, indicating a strong association compared with that in other age groups.

### 4.3. Limitations

This study had several limitations. First, CPR quality was only evaluated based on chest compression observed after EMT arrival at the scene and not during the whole bystander CPR period. Furthermore, only the last rescuer could be evaluated in the case of multirescuers. Second, the measurement of quality could not include quantified values for the rate and depth of compression, recoil of chest, and hands-off time. Because CPR quality was evaluated by visual observation of EMTs, there was a lack of objective evaluations. Third, there was a lack of bystander information such as history of CPR training and physical status. In addition, considerable information on the sex of the bystander was missing; thus, the impact of the sex of the bystander needs further investigation.

## 5. Conclusion

The main factor affecting CPR quality in this study was the age of the bystander, as older bystanders found it more difficult to maintain CPR quality. In addition, high-quality CPR had a greater effect on neurological recovery in older patients than in younger patients. To improve the quality of bystander CPR and the neurological prognosis of patients, CPR training among older people should be strengthened.

## Figures and Tables

**Table 1:** General characteristics and resuscitation variables according to bystander CPR quality.

	All *n* = 2,491	High-quality *n* = 149	Low-quality *n* = 2,342	*p* value	Cases with missing data

*Patients element*					
Sex, male	1,661 (66.7)	115 (77.2)	1,546 (66.0)	0.005	
Age, year (median, IQR)	70 (56–79)	62 (51.5–75.0)	70 (56–80)	<0.001	

*Medical history*					
Hypertension	763 (30.6)	33 (22.1)	730 (31.2)	0.022	
Diabetes mellitus	534 (21.4)	34 (22.8)	500 (21.3)	0.681	
Cerebrovascular disease	187 (7.5)	4 (2.7)	183 (7.8)	0.016	
Pulmonary disease	65 (2.6)	3 (2.0)	62 (2.6)	1.000	
Heart disease	424 (17.0)	15 (10.1)	409 (17.5)	0.018	
Malignancy	211 (8.5)	9 (6.0)	202 (8.6)	0.361	
Initial shockable rhythm	528 (21.2)	53 (35.8)	475 (20.3)	<0.001	3
Location, home	1,990 (79.9)	88 (59.1)	1,902 (81.2)	<0.001	
Witnessed	1,267 (51.2)	91 (61.5)	1,176 (50.5)	0.011	14

*System element*					
DACPR	2,338 (94.8)	136 (92.5)	2,202 (95.0)	0.182	25
RTI, min	7 (6–9)	7 (6–7)	7 (6–9)	0.131	

*Bystander element*					
Sex, male	910 (50.5)	46 (52.9)	864 (50.4)	0.662	689
Age group					
<40 years	383 (15.4)	35 (23.5)	348 (14.9)	<0.001	
40–49 years	546 (21.9)	42 (28.2)	504 (21.5)		
50–59 years	882 (35.4)	57 (38.3)	825 (35.2)		
≥60 years	680 (27.3)	15 (10.1)	665 (28.4)		
Relation with patient, family	2,101 (86.0)	106 (71.1)	1,996 (86.9)	<0.001	46
Multirescuers (≥2 rescuers)	1,064 (43.3)	84 (56.8)	1,312 (56.7)	1.000	31

*Outcome*					
Prehospital ROSC	590 (23.7)	62 (41.6)	528 (22.5)	<0.001	
Total ROSC	751 (30.1)	71 (47.7)	680 (29.0)	<0.001	3
Survival discharge	281 (11.3)	45 (30.2)	236 (10.1)	<0.001	8
Neurologically favorable discharge	172 (6.9)	38 (25.5)	134 (5.7)	<0.001	1

CPR, cardiopulmonary resuscitation; ROSC, return of spontaneous circulation; DACPR, dispatcher-assisted CPR; RTI, response time interval.

**Table 2:** Univariate and multivariate analyses of the influence of resuscitation variables on high-quality CPR performed by a bystander.

	OR (95% CI)	aOR (95% CI)
*Patients' elements*		
Age, year	0.98 (0.97–0.99)	1.00 (0.99–1.01)
Male	1.74 (1.18–2.58)	1.38 (0.91–2.09)
Occurrence at home	0.33 (0.24–0.47)	0.42 (0.27–0.64)
Witnessed	1.57 (1.11–2.20)	1.45 (1.03–2.06)

*Bystander elements*		
Relation with patient, family	0.37 (0.26–0.54)	0.77 (0.47–1.27)

*Age groups*		
<40 years	4.46 (2.40–8.28)	4.28 (2.24–8.17)
40–49 years	3.69 (2.03–6.74)	3.34 (1.80–6.176)
50–59 years	3.06 (1.72–5.46)	3.01 (1.68–5.39)
≥60 years	1	1

CPR, cardiopulmonary resuscitation; CI, confidence interval; OR, odds ratio; aOR, adjusted odds ratio.

**Table 3:** Association between the outcome and high-quality CPR according to the age of bystanders.

	Total	<40 years	40–49 years	50–59 years	≥60 years

Survival at discharge	2.84 (1.73–4.65)	5.25 (1.89–14.56)	1.22 (0.40–3.68)	2.67 (1.19–5.98)	4.42 (1.05–18.71)
Neurologically favorable discharge	4.29 (2.34–7.88)	4.26 (1.12–16.18)	3.43 (1.02–11.60)	5.55 (1.89–16.27)	7.61 (1.41–41.04)

Adjusted for sex, occurrence at home, shockable rhythm, witnessed status, and targeted temperature management. CPR, cardiopulmonary resuscitation.

## Data Availability

The SPSS data used to support the findings of this study are available from the corresponding author upon request.
